# Metastasis From Gastric Signet Ring Cell Adenocarcinoma Presenting as a Rectosigmoid Stricture: A Rare Case

**DOI:** 10.7759/cureus.8552

**Published:** 2020-06-10

**Authors:** Shehriyar Mehershahi, Nikhitha Mantri, Haozhe Sun, Danial Shaikh, Harish Patel

**Affiliations:** 1 Gastroenterology, BronxCare Health System, Bronx, USA; 2 Medicine/Internal Medicine, BronxCare Health System, Bronx, USA; 3 Medicine/Gastroenterology, BronxCare Health System, Bronx, USA; 4 Internal Medicine, BronxCare Health System, Bronx, USA; 5 Internal Medicine, Bronx Lebanon Hospital Center, New York, USA

**Keywords:** signet ring cell type carcinoma, gastric cancer, colonic stricture

## Abstract

We present the case of a 55-year-old man who was admitted with new abdominal ascites and change in stool caliber. The colonoscopy examination revealed a severe stricture with inflamed friable mucosa measuring 8 cm in length in the rectosigmoid colon. Histologically, poorly differentiated adenocarcinoma with signet ring cells was seen. The patient underwent esophagogastroduodenoscopy (EGD), which was suggestive of linitis plastica of the stomach. On microscopic examination, biopsy reported poorly differentiated adenocarcinoma with occasional signet ring cells as the primary source.

## Introduction

Gastric cancers are the third most common cause of cancer-related deaths worldwide [1]. Among the various gastric malignancies, signet ring cell carcinoma (SRC) is well-known to have an aggressive course and, therefore, poor prognosis. Linitis plastica is the macroscopic presentation of a diffuse infiltrating carcinoma of a hollow organ, causing it to retain its shape but remain stiff and contracted. A study by Piessen et al. had a total of 27 cases of linitis plastica, of which 21 had a histological diagnosis of SRC [2]. It is important not to consider linitis plastica and SRC as synonyms.

There have been very few cases of gastric SRC with metastasis to the large intestine presenting in the form of polyps, ulcerations, and depressed lesions. We present a case of gastric SRC with metastasis to the rectosigmoid junction presenting as a stricture.

## Case presentation

A 55-year-old male presented to our emergency room with left-sided, intermittent, dull, lower abdominal pain, and progressive abdominal distension of five months duration. The patient reported intermittent constipation with a change in stool caliber. He otherwise denied weight loss, loss of appetite, dark stools, and blood in stools. His medical conditions included hypertension, fatty liver, and gastroesophageal reflux disease. Surgical history was significant for umbilical and left inguinal hernia repair. He never used tobacco or recreational drugs. He had been consuming up to eight beers every day for many years and quit three months prior to his presentation. He denied the use of any over-the-counter medications or herbal medications. There was no history of gastrointestinal (GI) malignancies in the family. His medications included pantoprazole, atenolol, and stool softeners as needed.

**Figure 1:**
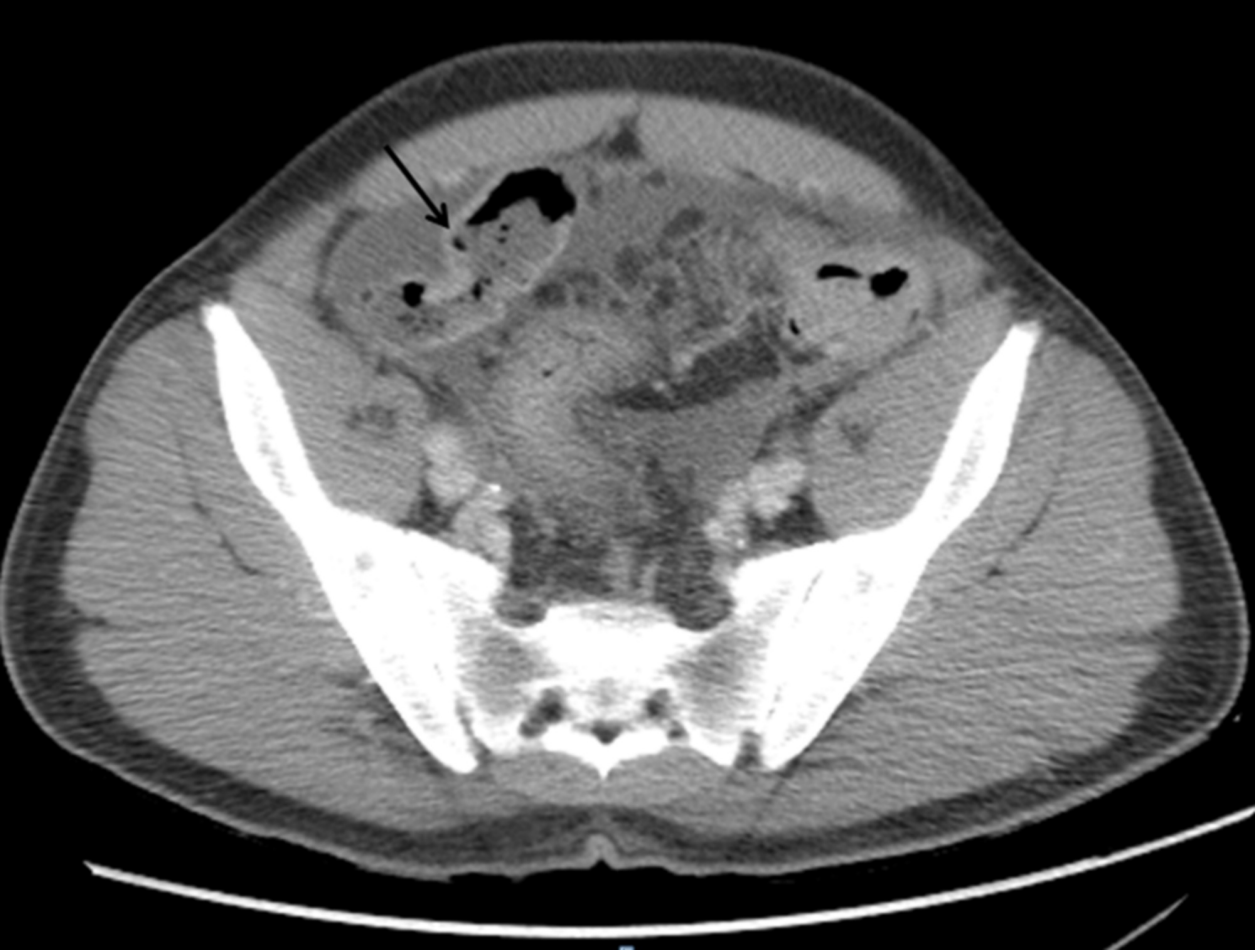
Computed axial tomography of the abdomen and pelvis showed rectosigmoid thickening

He presented to the emergency room with stable vitals. Physical examination was significant for mild abdominal distention, fluid trill, and shift. Laboratory findings revealed normal complete blood count and liver function tests. Computed tomography (CT) of the abdomen (Figure 1) showed mild wall thickening of the sigmoid colon and rectum. He was also noted to have a moderate-to-large amount of simple density ascites, a mildly enlarged liver, and a normal spleen.

**Figure 2:**
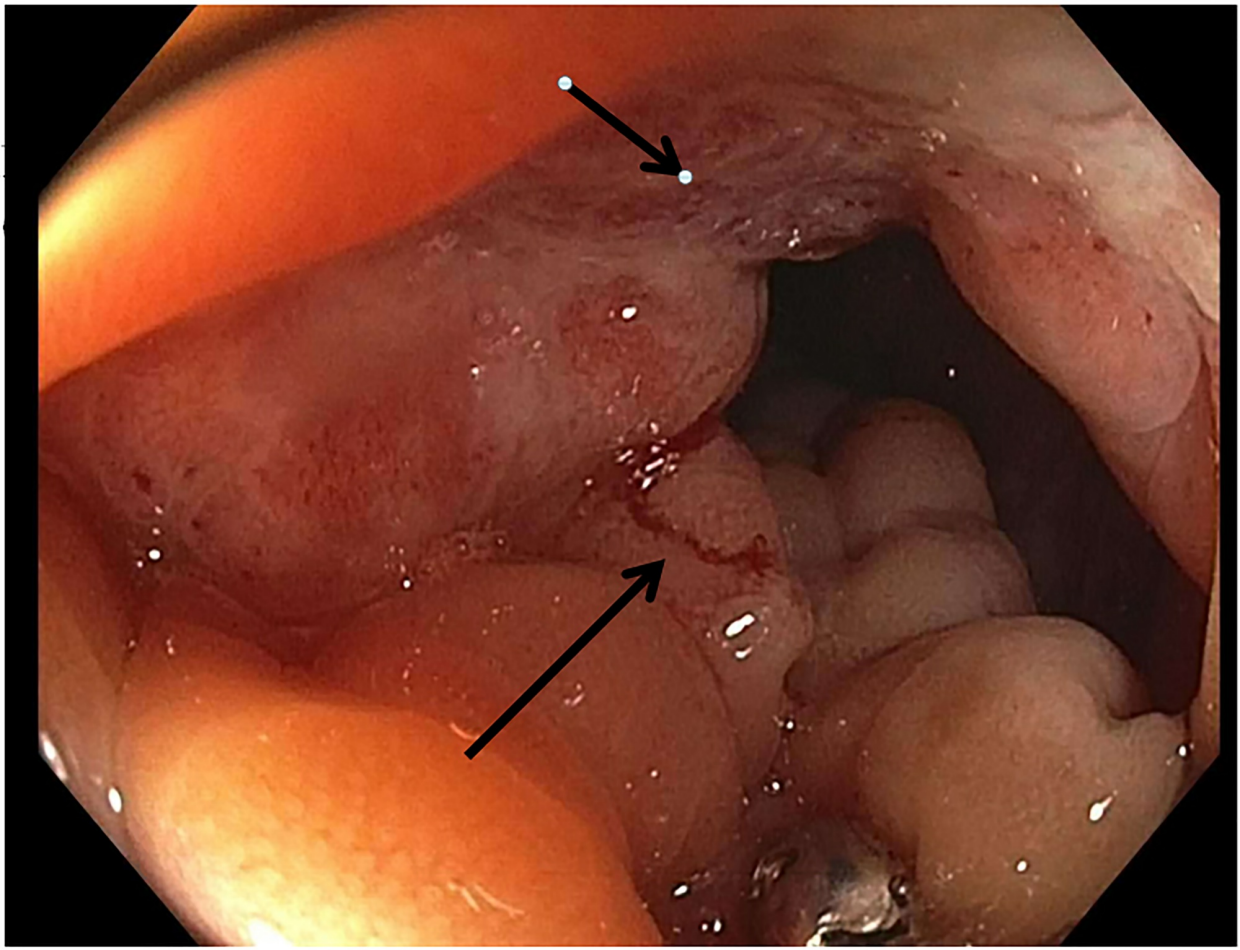
Colonoscopic view of the rectosigmoid colon stricture with inflamed friable mucosa

A diagnostic paracentesis showed cloudy fluid with a serum to ascites albumin gradient (SAAG) ratio of 1.4, lymphocytic predominance, and total protein of 4.4. Cytology revealed highly atypical epithelial cells, suspicious for carcinoma. The patient underwent colonoscopy, which revealed a severe stricture with inflamed friable mucosa measuring 8 cm in length in the rectosigmoid colon located 12-20 cm from the anal verge (Figure 2). The standard adult colonoscope was not able to be traverse due to severe stenosis; it was switched to an ultrathin scope, which was advanced with ease to the ileocecal valve. There were diffuse erythema and granularity in the descending, transverse, and ascending colon. Multiple biopsies were performed throughout the entire colon, including the stenotic lesion.

The pathology of the stenotic lesion was reported as a poorly differentiated adenocarcinoma with signet ring cells. The rest of the biopsies were reported to be colonic mucosa with preserved crypt architecture.

**Figure 3:**
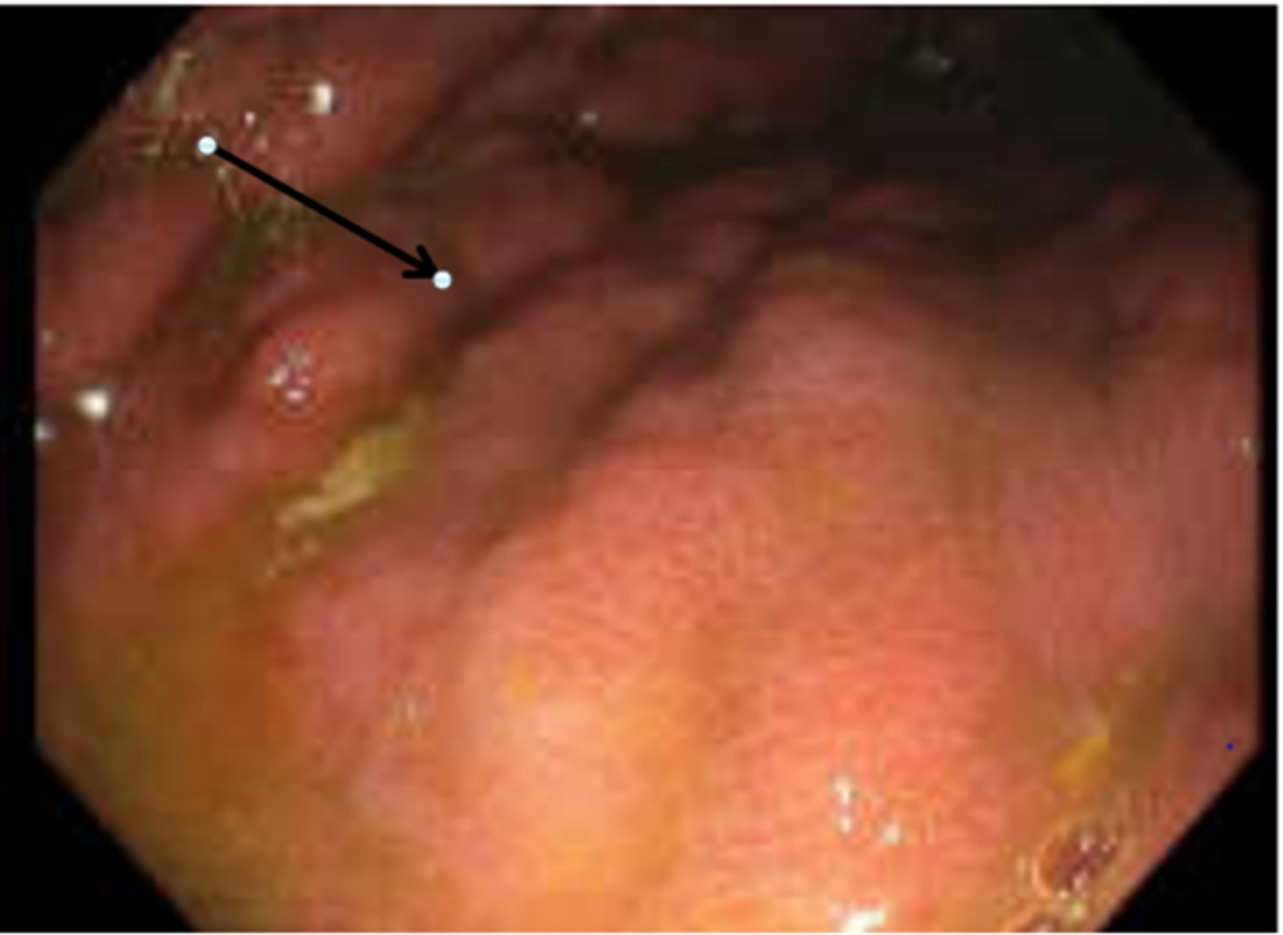
Upper endoscopic view showing the gastric fundus revealing thickened gastric folds with diffuse granular mucosa

The patient underwent esophagogastroduodenoscopy (EGD; Figure 3), which showed linitis plastica of the stomach with a non-bleeding gastric ulcer in the body. Multiple areas of the stomach were biopsied given the appearance of linitis plastic, which was reported to be poorly differentiated adenocarcinoma with occasional signet ring cells. CT of the chest did not reveal any metastasis. The patient was referred to oncology for further management and was lost to follow-up.

## Discussion

Gastric SRC is uncommon and tends to have poor survival. Histologically, SRC is primarily composed of more than 50% tumor cells with predominantly mucinous cytoplasm and an eccentrically located crescent-shaped nucleus [3]. There has been an overall decrease in the incidence of gastric cancers over the years; however, the incidence of SRC has been on the rise in the United States [4]. A review article by Antonioli DA showed a rise in SRC cases from 9% to 39% in their newer series on gastric cancers done from 1975-1978 as compared to their previous similar series from 1938-1942 [5]. Theuer et al. reported a histological pattern of SRC in 3% to 39% of gastric cancer cases [6].

SRC is known to have a female predominance, is more common in the younger population, and is usually located in the middle and distal stomach [5,7]. In addition, it is predominant among blacks, Asians, American Indians, and Hispanics [8]. Patients are usually diagnosed at late stages (American Joint Committee on Cancer (AJCC) Stages 3 and 4) or with nodal or distant metastasis, one of the reasons being the infiltrative pattern leading to the late onset of clinical symptoms [8].

SRC occurs primarily in the stomach with common sites of metastasis being the lymph nodes, peritoneum, and intestines. Most of the time, when there is a large intestinal metastasis, the primary malignancy is usually the stomach [9]. Our case has been one among such where the patient was found to have ascites and a rectosigmoid stricture, which led to further investigation and tracing of the primary malignant lesion to the stomach. The literature review revealed various macroscopic presentations of the metastatic intestinal lesions. They included mostly polyps [10-13], ulcerations [14-15], and depressed lesions [9,16-17]. However, literature on metastatic lesions of a gastric SRC presenting as a colonic stricture is rare.

Another unique feature of our case is the location of SRC metastasis. A literature review done by Sonoda et al. showed 11 cases of primary gastric SRC with metastasis to the large intestine, out of which six cases had metastasis to the sigmoid colon and only one case of metastasis to the rectum [9]. Our patient had an uncommon metastasis to the rectosigmoid junction [18-19].

## Conclusions

Gastric SRC metastasis to the rectosigmoid junction in the form of stricture formation is a rare presentation. The literature review has revealed mostly gastric SRC metastasis to the colon as ulcerations or depressed or flat, elevated lesions. Given the rarity of gastric SRC metastasis to the colon with stricture formation at the rectosigmoid junction, this case helps build awareness and increase knowledge among physicians to look for possible gastric SRC in patients with colonic strictures.
